# PathwayVoyager: pathway mapping using the Kyoto Encyclopedia of Genes and Genomes (KEGG) database

**DOI:** 10.1186/1471-2164-6-60

**Published:** 2005-05-03

**Authors:** Eric Altermann, Todd R Klaenhammer

**Affiliations:** 1 Department of Food Science, Box 7624, North Carolina State University, Raleigh, NC 27695-7624, USA

## Abstract

**Background:**

Equally important and challenging as genome annotation, is the subsequent classification of predicted genes into their respective pathways. The Kyoto Encyclopedia of Genes and Genomes (KEGG) represents a database consisting of known genes and their respective biochemical functionalities. Although accessible online, analyses of multiple genes are time consuming and are not suitable for analyzing data sets that are proprietary.

**Results:**

Presented here is a new software solution that utilizes the KEGG online database for pathway mapping of partial and whole prokaryotic genomes. PathwayVoyager retrieves user-defined subsets of the KEGG database and stores the data as local, blast-formatted databases. Previously selected datasets can be re-used, reducing run-time significantly. Whole or partial genomes can be automatically analyzed using NCBI's BlastP algorithm and ORFs with similarities below the user-defined threshold will be marked on pathway maps. Multiple gene hits are sorted by similarity. Since no sequence information is transmitted over the Internet, PathwayVoyager is an ideal solution for pathway mapping and reconstruction of confidential DNA sequence data.

**Conclusion:**

PathwayVoyager represents an alternative approach to many already existing, more complex pathway reconstructions software solutions. This software does not require any dedicated hardware or software and is flexible and straightforward to use. It is ideally suited for environments where analyses on variable datasets are desired.

## Background

The ongoing sequencing of complete genomes of prokaryotes and eukaryotes reveals a tremendous amount of uncharted data. In prokaryotic genomes, roughly 25 to 30 percent of the predicted ORFeome remain functionally unknown with many Open Reading Frames (ORFs) only showing similarities to conserved hypothetical ORFs of other organisms. However, a significant number of the predicted ORFs do show similarities to functionally classified genes with defined roles in the complex network of metabolic pathways. Previously, the most common approach to determine the functionality of a gene and its gene product was to experimentally determine phenotypic changes upon inactivation or overexpression of the gene. This is the most effective approach for determining the roles of single genes, but it is unfeasible to investigate complete ORFeomes with over 2000 ORFs. The Kyoto Encyclopedia of Genes and Genomes (KEGG) represents an ambitious and successful attempt to assign known enzymes into known biochemical pathways and is updated on a regular basis [1-3]. The database is represented by a web-based browser and a multitude of different analyses are possible. Genes can be analyzed using online Blast algorithms  and if significant Blast similarities resulted in the assignment of a defined enzyme class (i.e. EC classification), these genes can be marked in corresponding KEGG pathways. However, analysis of larger numbers of genes using this manual approach is tedious, inflexible, and time consuming. Since un-encrypted data transmission is used for most public servers, remote Blast analyses with confidential sequence queries is not desirable. In addition to this web-browser based approach, the KEGG database can also be accessed directly via an application programming interface (API) and the underlying databases can be downloaded for local uses. Third party-software most commonly use the KEGG database for gene-classifications, often in combination with whole genome annotation efforts [4], or utilizes the database content for reference gene sets used in further experiments [5]. Other remote software solutions like DAVID [6] integrate various databases and experimental results to allow for extensive query-based data mining on given gene lists. Similarly, Pathway Tools  utilizes dedicated server and database backbones to realize a sophisticated environment. New genomes must be manually integrated into a Pathway/Genome Database (PGDB), which in turn sets the basis for more complex queries and analyses. However, the significant resources required to implement genome information for Pathway Tools might not be readily available. PathFinder [7] and BioMiner [8] use a different approach in that they utilize some of the data the KEGG database provides and then approach pathway reconstruction using software specific algorithms. However, these solutions tend to be complex, thoroughly web-based, and often utilize the whole KEGG databases without selective options. Furthermore, most of these solutions require specific data formats that are compatible with the respective application for initial data parsing and entry. For example, although PathFinder can parse common EMBL input files, only genes with an EC-number tag can initially be integrated into the database system, implying a sophisticated level of existing genome annotation. Although this approach can be used to generally classify genes, it lacks the necessary flexibility to compare specific pathways of interest in single organisms or groups of organisms to a selected set of genes. In addition, these complex algorithms are not always needed and more simplistic and faster solutions with less hardware and software requirements would be preferable due to their ease of use and flexibility.

Closely-related groups of organisms may differ in certain key elements that define specific strain/species related differences. Comparing these organisms or groups of organisms with each other should highlight these differences and reveal specific properties or lead to new genetic targets for pathway engineering. Therefore, it may be desirable to choose only subsets of organisms and pathways from the overall KEGG database content.

PathwayVoyager was developed to overcome most of these obstacles. The software resembles a tool to analyze an unlimited number of protein sequences against a user-selected subset of the KEGG database using NCBI's BlastP algorithm and subsequently places them into their proper pathway positions. Results are displayed in colored pathway maps and hits can easily be analyzed using the graphical interface. This tool reflects a different approach to pathway mapping, in that it provides a simplistic and flexible approach with few prerequisites. No dedicated hardware (i.e. background server) or software (i.e. relational database backbones) are necessary to analyze given datasets. A standard PC with the Windows operating system is sufficient to operate PathwayVoyager. In contrast to more complex tools, no underlying protein annotation is necessary and plain protein sequences in FASTA format can be used as query templates. This approach is ideal for draft phase genomes and ongoing annotation efforts in completed genomes where the emphasis lies on the establishment and verification of gene annotation and an initial assessment of metabolic capabilities. The resulting main advantage of PathwayVoyager is its speed and economy for initial pathway mapping. Also, the resulting data can easily be accessed on different locations by transferring the generated flatfile database to the respective computers. Once the research objective shifts to comparative and predictive pathway analyses, other tools like DAVID or PathwayTools become more advantageous. PathwayVoyager fills a niche for environments with limited hardware and software resources that still require a significant and meaningful way to perform small and large scale pathway mapping projects from varying data sources.

## Implementation

PathwayVoyager is written completely in Perl/Tk and requires the Perl interpreter . No further Perl modules are required. However, two external distributions are required, namely the NCBI Blast distribution , and the SOAP::Lite client  to utilize the KEGG API. Perl/Tk provides the interpreter for PathwayVoyager, and the SOAP::Lite client facilitates interaction with the KEGG API. The standalone Blast distribution is used to generate Blast compatible databases and to perform the local Blast analyses. For data analysis and browsing, the Perl/Tk interpreter is the only pre-requisite for PathwayVoyager. The standalone Blast distribution, and the SOAP::Lite client can be omitted and no internet connection is necessary.

The software was developed to optimally complement the GAMOLA annotation suite [9] but accepts any protein sequence in FASTA format. The sequential numbering of ORFs in GAMOLA annotated genomes is reflected by gene-name tags in the generated Genbank files. Extracting protein sequences into FASTA files preserves this numbering scheme and is subsequently presented in the browser module of PathwayVoyager. This permits fast and efficient ORF-tracking throughout the genome and often provides preliminary identification of gene clusters. PathwayVoyager operates as a stand alone software solution without the need of additional database backbone systems. The use of PathwayVoyager and the KEGG database system implies the agreement to the license terms specified for KEGG at .

**Figure 1:**
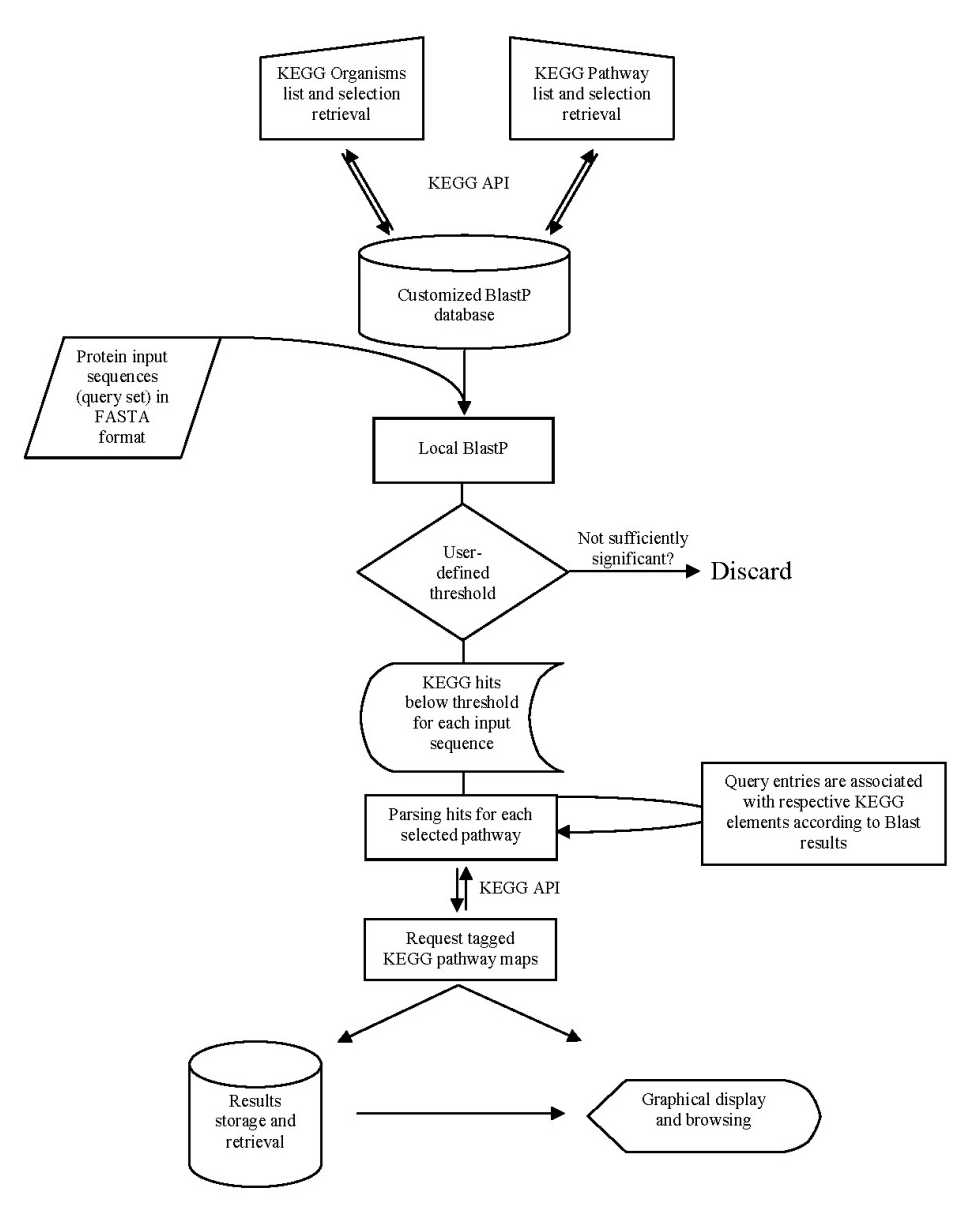
**Pathway Voyager mapping procedure**. The analysis and mapping procedure of PathwayVoyager is shown in a flowchart diagram. Manual selection of organisms and pathways present in the KEGG database, at the time of analysis, results in the retrieval of a specific set of protein sequences that are subsequently reformatted into a BlastP database. Protein query sequences are then used as templates for local BlastP analyses. Results are subjected to a user-defined threshold and subsequently parsed to retrieve tagged KEGG pathway maps. Pathway graphs and parsed BlastP results are stored as a flatfile database and can be displayed using the graphical browser. Opposite double arrows represent Internet-access using the KEGG API. Symbols used are according to general flowchart conventions.

The general procedure used by PathwayVoyager to map genes is illustrated in Figure 1. User defined organism and pathway subsets of the KEGG database at the time of analysis result in the online retrieval of a defined set of protein sequences via the KEGG API (API methods: list_organisms; list_pathways). This dataset is converted into a BlastP compatible database using "formatdb" provided by NCBI. The query protein sequences in FASTA format are then subjected to a BlastP analysis, utilizing "blastall", also part of the standalone Blast distribution. Blast results for each query are formatted and saved into a flatfile database, linking each query to KEGG EC-number entries. KEGG map points are then requested for each selected pathway (API methods: get_enzymes_by_pathway, get_genes_by_pathway,) and stored in an internal tabular format. Subsequently, the Blast flatfile database is analyzed and Blast hits above a user-defined e-value threshold are discarded. The remaining significant Blast hits are then parsed to group query-sequences to KEGG map points for each selected pathway. Each KEGG entry in a given pathway is assigned to a list of significant Blast hits. Each hit displays the respective query ORFs and its best Blast hit based on the selected KEGG database subset, and the corresponding Blast scores and e-values. Based on this query-assignment, tagged KEGG pathway graphs are requested online using the KEGG API (API method: mark_pathway_by_objects). Resulting GIF maps and ASCII group assignment data files are stored in a flatfile database and can be visualized using the graphical browser module. Previous analyses can be accessed directly from the browser without the need to re-analyze the query sequences.

The graphical user interface was designed to be self-explanatory and easy to use. After the initial pathway setup, no further installation steps are necessary. Although PathwayVoyager requires an internet connection in order to retrieve data from the KEGG database, all analyzes involving the provided gene sets are performed locally and no sensitive data are transmitted. This eliminates one of the major security concerns when working with confidential data and permits the real-time use of the KEGG database system. PathwayVoyager does not require any dedicated hardware and has been tested on a standard PC and the Windows platform. Linux versions of Perl/Tk, the SOAP::Lite client, and the standalone Blast distribution are freely available and would allow PathwayVoyager to operate under a Linux environment, as well. For certain selectable pathways (e.g. Ribosomal reference pathway) KEGG does not yet support organism independent marking. For practical reasons, no hits will be displayed for these pathways.

## Results and discussion

**Figure 2:**
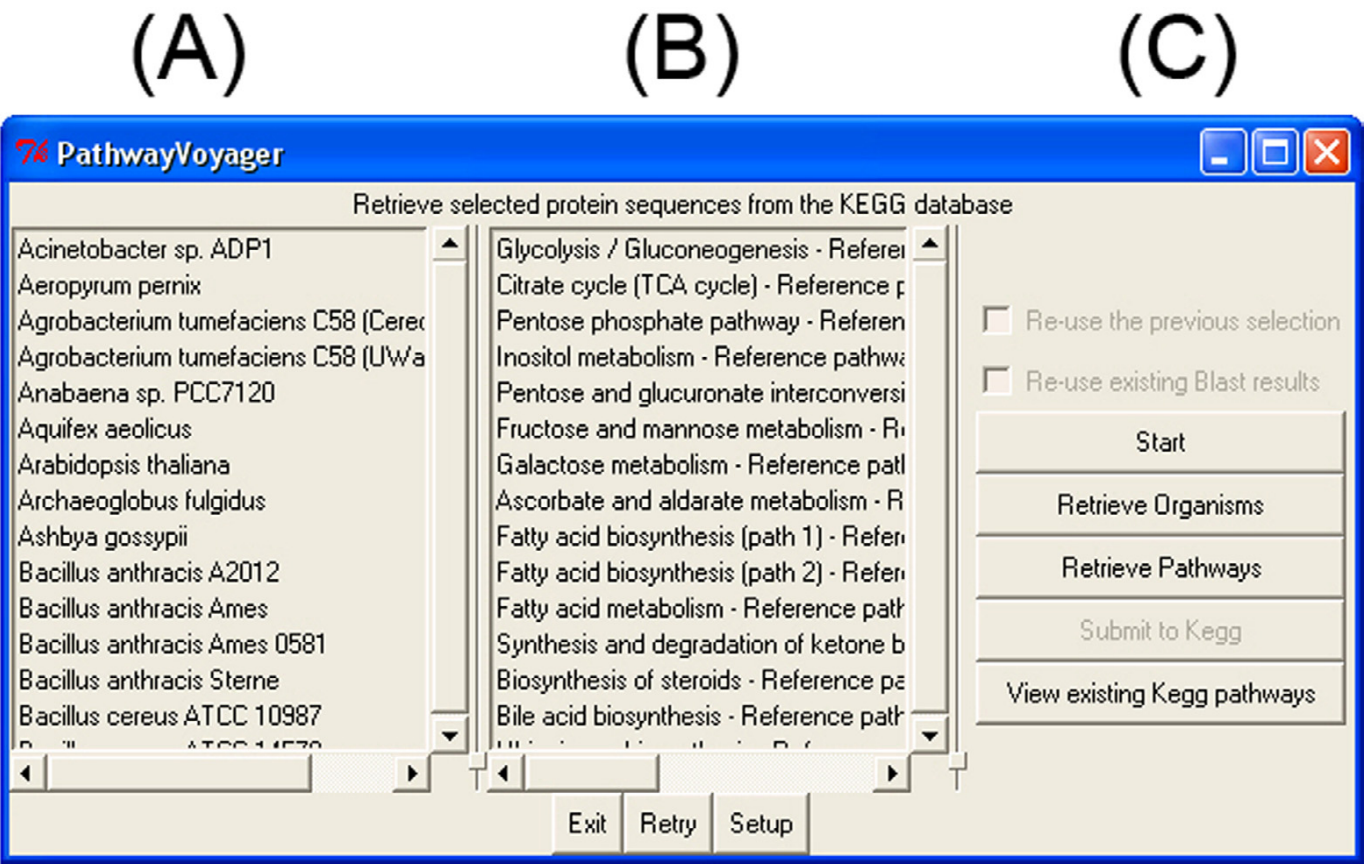
**PathwayVoyager main window**. Screenshot of the graphical user interface. (A) and (B) indicate listboxes showing the organisms that can be individually selected and pathways represented in KEGG at the time of analysis. Button for exiting the software, redo the analysis, and accessing the setup are located beneath both boxes. Section (C) resembles the step-by-step design to start the analysis, retrieve the selected organisms and pathways, and subsequently obtain the respective protein sequences. In manual mode, the KEGG analysis and pathway retrieval can be activated by the user. Existing KEGG analyses can be reviewed by accessing the lowest button. On-the-fly options, for re-using previous organism-pathway selections and respective BlastP results, are located in the top region of section (C).

Upon start, the main window displays two list-boxes and the currently active buttons (Figure 2, A, B, and 2C, respectively). Changing the default values is possible at this stage through the "Setup" function and has to be done before initializing the analysis. The "Start" button (Figure 2, C) commences the analysis and all organisms currently present in the KEGG database will be displayed in a listbox (Figure 2, A). Single and multiple selections are possible and will be confirmed with the "Retrieve Organisms" button. The second listbox (Figure 2, B) will then be automatically populated with KEGG pathways. After selecting the desired pathways to be analyzed, the "Retrieve Pathways" button confirms the selection and starts the KEGG pathway mapping. Both, the organisms and the pathways present in the KEGG database at the time of the analysis can be selected independently from each other, with the exception of organism-specific pathways (i.e. ABC transporter or two-component regulatory systems). This guarantees use of the most flexible solution for selective comparative analyses between groups of organisms. By selecting all organisms and pathways, the given gene set can be compared against the complete KEGG database.

The user-selected organism and pathway combination is shown in a separate pop-up window (not shown). The current status of the KEGG pathway mapping is also shown in a separate log-window (not shown). In general, the right panel (Figure 2, C) harbors the user-guide interface and was designed to lead the user through the analyses in a step-by-step approach. By default, the organism and pathway confirmation automatically initializes the KEGG pathway mapping. If only the selected and retrieved protein sequences are required, or a manual start for pathway mapping is desired, the setup module allows the configuration for manual mode. KEGG pathway mapping can then be initiated with the "Submit to KEGG" button. Selected pathway/organism combinations are saved as an ASCII text file. The retrieved protein sequences are stored into a separate ASCII file and a Blast-compatible database is generated. For future analyses, the pathway/organism selection and the respective database can be re-used with different query protein sequence sets.

The possibility to re-use previous selections dramatically reduces the time needed to complete KEGG analyses, as retrieval of individual protein sequences from KEGG is omitted. In addition, Blast results obtained with the given query set can also be re-used. This shortens the run time further, enabling rapid mappings and analyses of pathways with varying relaxed or stringent threshold values.

The provided gene set will then be compared to the local database generated from the selected organism-pathway protein sequence combination using the BlastP algorithm. Blast hits featuring an e-value below the user-selectable threshold will be used to generate the marked KEGG pathway requests. Pathway maps are saved as GIF files and the URL for the respective KEGG pathway map including the corresponding BlastP results are stored separately in text files.

**Figure 3:**
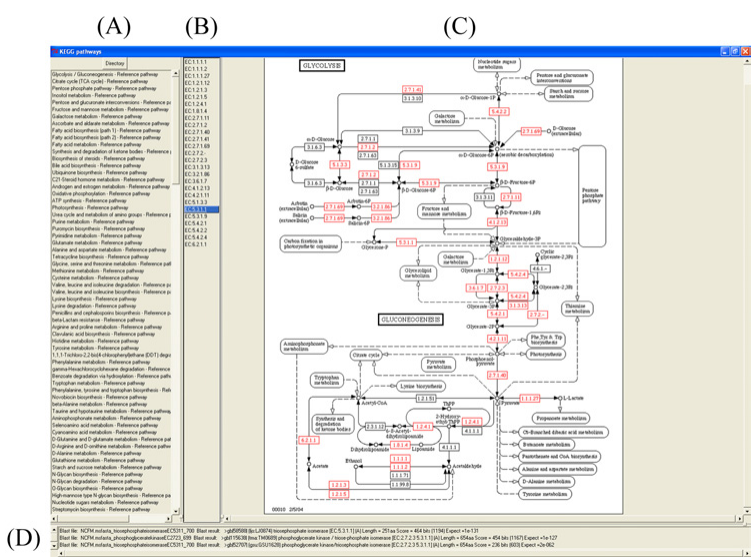
**Interactive KEGG Pathway display**. The screenshot illustrates KEGG pathway mapping for the glycolysis/gluconeogenesis pathway using the predicted ORFeome of the GAMOLA annotated *L. acidophilus *NCFM genome as query template. The e-value threshold was set to 1e-10. Section (A) shows all pathways used for this analysis. Section (B) lists the marked entries. Section (C) represents the graphical representation of a selected pathway. Elements exhibiting BlastP hits below the selected threshold e-value are marked as red boxes. Section (D) shows the corresponding BlastP results, comprising the respective query protein designation, the corresponding KEGG hit, its amino-acid length, the BlastP-score, and e-value. Entries are sorted by ascending e-values.

Results are displayed in a separate window. Figure 3 illustrates pathway mapping for the Glycolysis/Gluconeogenesis pathway (KEGG pathway code: 00010) using the ORFeome of *L. acidophilus *NCFM [10] as query set and the complete KEGG database as template.

In general, previously selected pathways are displayed by either their KEGG pathway code or full name. Alternative analyses can be displayed by changing the default mapping directory, using the "Directory" function (Figure 3, A). The selected pathway will then be graphically displayed and BlastP hits below the specified threshold are indicated as red boxes, bearing the respective EC numbers (Figure 3, C). Each marked element is shown by its EC-number code, numerically sorted, in a listbox (Figure 3, B). Upon selection of an entry, all BlastP hits below the threshold are sorted by ascending e-values and displayed accordingly (Figure 3, D). This workflow allows for a quick pathway mapping throughout a given gene set and those potentially involved in multiple pathways can be easily identified and analyzed.

In the example shown, the conversion of glyceronephosphate to glyceraldehyde-3-phosphate is mediated by a triosephosphate isomerase (EC 5.3.1.1). Selecting this entry from the EC entry list (Figure 3, B), highlights all query hits found in *L. acidophilus *below the defined threshold (Figure 3, D). Two entries below an e-value of 1e-120 were found, namely ORFs Lba699 (e-value: 1e-127) and Lba700 (e-value: 1e-131). Both entries show significant similarities to triosephosphate isomerases. Further analyses showed that the conversion of glyceraldehyde-3-phosphate to glycerate-1,3-bisphosphate and to glycerate-3-phosphate is mediated by Lba698 (EC 1.2.1.12, e-value 1e-176) and Lba699 (EC 2.7.2.3, e-value 0), respectively. The ambiguity found for EC 5.3.1.1 could be resolved and, consequently, the genome annotation was updated accordingly. More detailed analyses revealed the presence of the complete pathway for uptake and conversion of glucose into pyruvate and L-lactate. A more detailed analysis of the complete metabolic pathway reconstruction of *L. acidophilus *NCFM using PathwayVoyager is described elsewhere [10].

PathwayVoyager does not evaluate or extrapolate the displayed hits and the quality and significance of the results depend on the current content of the KEGG database. As with every predictive software, results should be carefully analyzed and seen in their genetic context to evaluate activities and potential substrate specifity-variances carried out by homologous enzymes. Results from previous analyses can be displayed by selecting the "View existing KEGG pathways" option in the PathwayVoyager main window (Figure 2, C).

Run times for PathwayVoyager may vary, depending on the number of selected pathways and organisms. Analysis of a complete genome of ~2,000 open reading frames (ORFs) using the complete KEGG database can be carried out in less than 36 h.

## Conclusion

PathwayVoyager differs significantly in its approach from other software solutions for pathway reconstructions that already exist. In contrast to the often highly complex and specific algorithms, PathwayVoyager represents a more straight-forward approach, and doesn't require substantial resources on the users' side. Relying on the Blast algorithm and the ambitious KEGG database, PathwayVoyager utilizes widely accepted resources to analyze and map data. Despite the uncomplicated approach, evidential data can rapidly be obtained and easily analyzed during genome analyses. PathwayVoyager represents an effective pathway mapping tool for large or confidential data sets.

## Availability and requirements

• **Project name: **Biological Pathway Mapping

• **Project home page: **none

• **Operating system(s): **Platform independent

• **Programming language: **PERL

• **Other requirements: **Active Perl 5.8, SOAP::Lite client, NCBI's Blast distribution

• **License: **The software is distributed for free under the NC State University copyright and can be obtained upon request to the authors.

• **Any restrictions to use by non-academics: **none

## Authors' contributions

EA developed and tested the complete PathwayVoyager software and performed the described pathway reconstruction for *Lactobacillus acidophilus *NCFM. TRK read and approved the manuscript and provided financial support for EA and the project.
